# Entangled Qubit States and Linear Entropy in the Probability Representation of Quantum Mechanics

**DOI:** 10.3390/e24040527

**Published:** 2022-04-09

**Authors:** Vladimir N. Chernega, Olga V. Man’ko, Vladimir I. Man’ko

**Affiliations:** 1 Lebedev Physical Institute, Russian Academy of Sciences, Leninskii Prospect 53, 119991 Moscow, Russia; chernegavn@gmail.com (V.N.C.); mankovi@lebedev.ru (V.I.M.); 2 Russian Quantum Center, Skolkovo, 143025 Moscow, Russia

**Keywords:** probability distributions, Bell states, qubits, symplectic tomogram, linear entropy

## Abstract

The superposition states of two qubits including entangled Bell states are considered in the probability representation of quantum mechanics. The superposition principle formulated in terms of the nonlinear addition rule of the state density matrices is formulated as a nonlinear addition rule of the probability distributions describing the qubit states. The generalization of the entanglement properties to the case of superposition of two-mode oscillator states is discussed using the probability representation of quantum states.

## 1. Introduction

The conventional description of system states in quantum mechanics is provided using either wave functions [1] (pure states) or density matrices [2,3] (mixed states). In addition, the vectors in the Hilbert space or the density operators acting in the Hilbert space are used to describe the pure states and the mixed states, respectively [4]. Different other representations of quantum states including the Wigner quasidistribution [5], the Husimi function [6], the Glauber–Sudarshan quasiprobability distribution [7,8], and the groupoid picture of quantum mechanics [9] were introduced to describe the systems with continuous variables such as oscillators. In the case of systems with discrete variables such as spin systems, the states were also described by analogs of the Wigner function of the discrete variables; see, e.g., [10]). The described representations of quantum states are very different from the representations used in classical physics, e.g., in classical statistical mechanics where the probability distributions are associated with the classical system states. The attempts to find the probability representation were done, e.g., in [11]).

Recently, the probability representation of quantum states was suggested for the systems with continuous variables in [12] and with discrete variables in [13,14,15]. In classical mechanics, the tomographic probability distribution of system states was introduced in [16]. All the representations of quantum states including wave functions, density operators, quasidistributions such as the Wigner functions, and the tomographic probability distributions contain equivalent information on the physical properties of the quantum states. However, the probability distribution description of the states sometimes gives the possibility to obtain a better understanding of the physical phenomena based on an intuition available due to the classical experience. The classical and quantum aspects of tomography were discussed in [17], and classical–quantum dynamics were considered in [18]. Recently, the probability representation of quantum states was used to study properties of cosmology [19,20]. The density matrices of qudit states were discussed in different representations in [21,22]. The application of the symplectic tomography scheme to the stimulated Raman scattering (SRS) process was provided in [23]. The tomographic probability distribution method was developed, and different aspects of its applications are presented in [24]. The review of the harmonic analysis of the density matrix properties including the method of symplectic tomography is given in [25]. The connection of the symplectic tomography method with the metaplectic group and the Radon transform was discussed in [26]. The tomographic causal analysis of two-qubit states was done in [27].

In the probability representation, the system states are described by the standard conditional probability distributions, which can be used to obtain all the other mentioned functions determining quantum states. For example, the Wigner function is connected with the tomographic conditional probability distribution [12] by means of the invertible integral Radon transform [28]. The review of constructing the probability representation of quantum states is presented in [29,30]. In quantum mechanics, the phenomenon of the entangled states discovered by Schrödinger [31] plays an important role.

The aim of this paper is to study the properties of this phenomenon on an example of qubit states, using the probability representation of the states introduced in [32,33]. The entanglement properties of qubit states were discussed by Bell [34]; in this paper, we consider the entanglement phenomena in the probability representation of quantum mechanics on an example of Bell states of qubits—the two spin-1/2 systems. The entanglement of the system pure states is associated with such characteristics as the linear entropy. In addition, the von Neumann entropy is used to characterize this phenomena. We also consider the linear entropy expressed through the probabilities (associated with probability distributions) to describe the properties of qibut and ququart quantum states.

This paper is organized as follows.

In Section 2, we review the method of constructing the probability representation of quantum states on the example of spin-1/2 (qubit) states. In Section 3, we present the superposition principle of pure qubit states formulated for density matrices of the orthogonal states following the approach suggested in [35]. In Section 4, we consider density operators of the states constructed either for systems with two qubit subsystems or for systems without subsystems and consider the entangled two-qubit states in the probability representation of quantum mechanics, using the known probability representation of each qubit state. In Section 5, we study the entangled Bell states for the system with two qubit subsystems. In Section 6, we discuss the notion of entanglement phenomenon for systems of two qubits and study the linear entropy of the entangled qubit states. In Section 7, we consider the entangled states for systems with continuous variables on the example of two-mode Gaussian states of quantum systems; the linear entropy of such system is discussed. We present our conclusions and prospectives in Section 8. Explicit formulas for the state vectors and density matrices expressed through the probabilities of the first and second spin projections on *x*, *y*, and *z* axes for two-qubit states are provided in the Appendix A.

## 2. The Probability Representation of Qubit States

The construction of the probability representation of quantum states is based on the Born rule [36,37]. The Born rule was discussed in the connection with the relation to classical probability theory in [38]. Namely, for two arbitrary density operators ${\hat{\rho }}_{1}$ and ${\hat{\rho }}_{2}$, the number $\mathrm{Tr}\left({\hat{\rho }}_{1}{\hat{\rho }}_{2}\right)=p_{\left(1\right)}^{\left(2\right)}$ is a non-negative number $0\leq p_{\left(1\right)}^{\left(2\right)}\leq 1$, which has the physical meaning of the probability to obtain the properties of the second state with the density operator ${\hat{\rho }}_{2}$, if one measures these properties in the system state with the density operator ${\hat{\rho }}_{1}$ (and vice versa). For pure states ${\hat{\rho }}_{1}=|\psi_{1}\rangle \langle \psi_{1}|$ and ${\hat{\rho }}_{2}=|\psi_{2}\rangle \langle \psi_{2}|$, the Born rule means that in the state with the state vector $|\psi_{1}\rangle$, the measured properties of the state with the state vector $|\psi_{2}\rangle$ have the probability given by the number $p_{\left(1\right)}^{\left(2\right)}={\left|\langle \psi_{1}|\psi_{2}\rangle \right|}^{2}$. Due to this property of the Born rule, the idea of constructing the probability representation of quantum states of any system either with continuous variables such as an oscillator or discrete variables such as a spin-1/2 (qubit) is as follows.

We discuss this idea on the example of qubit. The density matrix ρ of the qubit state is determined by the state density operator $\hat{\rho }$ acting in the Hilbert space H, a linear space with three basis vectors $|\psi_{x}\rangle ,\;|\psi_{y}\rangle ,\;|\psi_{z}\rangle$, which are eigenvectors of the spin projection operators $s_{x}=\frac{1}{2}\sigma_{x},\;s_{y}=\frac{1}{2}\sigma_{y},\;s_{z}=\frac{1}{2}\sigma_{z}$, where $\sigma_{x},\;\sigma_{y},\;\sigma_{z}$ are the Pauli matrices, and we assume the Planck constant ℏ=1. This means that the spin projection operators have the matrices

(1) $$s_{x}=\frac{1}{2}\left(\begin{array}{cc} 0 & 1\\ 1 & 0 \end{array}\right),\;s_{y}=\frac{1}{2}\left(\begin{array}{cc} 0 & -i\\ i & 0 \end{array}\right),\;s_{z}=\frac{1}{2}\left(\begin{array}{cc} 1 & 0\\ 0 & -1 \end{array}\right).$$

Thus, one can check that the normalized state vectors $|\psi_{x}\rangle ,\;|\psi_{y}\rangle ,|\psi_{z}\rangle$ read

(2) $$|\psi_{x}\rangle =\frac{1}{\sqrt{2}}\left(\begin{array}{c} 1\\ 1 \end{array}\right),\;|\psi_{y}\rangle =\frac{1}{\sqrt{2}}\left(\begin{array}{c} 1\\ i \end{array}\right),\;|\psi_{z}\rangle =\left(\begin{array}{c} 1\\ 0 \end{array}\right).$$

The corresponding pure-state Hermitian density matrices of the states with the state vectors (2) have the form

(3) $$\begin{array}{c} \rho_{x}=|\psi_{x}\rangle \langle \psi_{x}|=\frac{1}{2}\left(\begin{array}{cc} 1 & 1\\ 1 & 1 \end{array}\right),\;\rho_{y}=|\psi_{y}\rangle \langle \psi_{y}|=\frac{1}{2}\left(\begin{array}{cc} 1 & -i\\ i & 1 \end{array}\right),\\ \rho_{z}=|\psi_{z}\rangle \langle \psi_{z}|=\left(\begin{array}{cc} 1 & 0\\ 0 & 0 \end{array}\right). \end{array}$$

An arbitrary 2×2 density matrix

$$\rho =\left(\begin{array}{cc} \rho_{11} & \rho_{12}\\ \rho_{21} & \rho_{22} \end{array}\right)$$

has the properties [3] ${\hat{\rho }}^{†}=\rho$, Trρ=1, i.e., $\rho_{11}+\rho_{22}=1$, and the eigenvalues of ρ are non-negative.

For the pure states $\rho_{\psi }=\left|\psi \rangle \langle \psi \right|$, one has the property $\rho_{\psi }^{2}=\rho_{\psi }$ and $\mathrm{Tr}\rho_{\psi }^{2}=1$. The idea of constructing the probability representation of quantum states of a spin-1/2 system can be formulated as follows.

It is possible to express three real parameters of an arbitrary density matrix ρ of the qubit state in terms of probabilities, which can be obtained using the Born rule. The minimal number of such independent probabilities is equal to three. The larger number can also be used, but to get three probability parameters, it is sufficient to calculate three probabilities given by three numbers

(4) $$p_{1}=\mathrm{Tr}\left(\rho \rho_{x}\right),\;p_{2}=\mathrm{Tr}\left(\rho \rho_{y}\right),\;p_{3}=\mathrm{Tr}\left(\rho \rho_{z}\right).$$

One can check that numbers $\rho_{11},\;\rho_{22},\;\rho_{21}=\rho_{12}^{*}$, and $\rho_{22}=1-\rho_{11}$ provide an explicit form of the qubit density matrix [32,33] in terms of the probabilities, namely,

(5) $$\rho =\left(\begin{array}{cc} p_{3} & p_{1}-\frac{1}{2}-i\left(p_{2}-\frac{1}{2}\right)\\ p_{1}-\frac{1}{2}+i\left(p_{2}-\frac{1}{2}\right) & 1-p_{3} \end{array}\right).$$

Thus, the spin-1/2 state density matrix is described by the probabilities of the measurable spin projections equal to +1/2 on three perpendicular directions of *x*, *y*, and *z* axes. We use the density matrices $\rho_{x},\;\rho_{y},\;\rho_{z}$ (3) of the pure spin states. However, all other state density matrices, including mixed state density matrices, can be also used to express the density matrix ρ parameters in terms of the probabilities given by the Born rule. Thus, the state of qubit is described by three dichotomic probability distributions given by the pairs of non-negative numbers $0\leq p_{i}\leq 1,\;i=1,2,3$, i.e., $\left(p_{1},1-p_{1}\right),\;\left(p_{2},1-p_{2}\right),\;\left(p_{3},1-p_{3}\right)$, which satisfy the condition of non-negativity of the eigenvalues of the density matrix ρ (5). It reads

(6) $$\sum_{j=1}^{3}{\left(p_{j}-\frac{1}{2}\right)}^{2}\leq \frac{1}{4}.$$

For pure qubit states, the probabilities $p_{j}$ satisfy the equality

(7) $${\left(p_{1}-\frac{1}{2}\right)}^{2}+{\left(p_{2}-\frac{1}{2}\right)}^{2}+{\left(p_{3}-\frac{1}{2}\right)}^{2}=\frac{1}{4}.$$

This means that the probabilities $p_{1},\;p_{2},\;p_{3}$ determine the points on the sphere of the radius equal to 1/2, which has the center position in the points $p_{1}=p_{2}=p_{3}=1/2$. For mixed states, the probabilities $p_{1}=p_{2}=p_{3}=1/2$ determine the points inside the sphere.

In fact, three dichotomic probability distributions can be considered as one conditional probability distribution, which we denote as Π(j|k), with j=1,2 and k=1,2,3 determining the quantum state of one spin-1/2 system. This notation means $\Pi \left(j=1|k=1\right)=p_{1}$, $\Pi \left(j=2|k=1\right)=1-p_{1}$, $\Pi \left(j=1|k=2\right)=p_{2}$, $\Pi \left(j=2|k=2\right)=1-p_{2}$, $\Pi \left(j=1|k=3\right)=p_{3}$, and $\Pi \left(j=2|k=3\right)=1-p_{3}$. This conditional probability distribution satisfies the relations

$$\sum_{j=1}^{2}\Pi \left(j|k\right)=1,\;0\leq \Pi \left(j|k\right)\leq 1.$$

Using the known Bayes formula for conditional probability distributions of two random variables, one can check that the probability distributions W(j,k) are given by

$$W\left(j,k\right)=\Pi \left(j|k\right)\tilde{P}\left(k\right).$$

Here, the function $\tilde{P}\left(k\right)$ is the probability distribution of random variable *k* satisfying the relations

$$\sum_{k=1}^{3}\tilde{P}\left(k\right)=1,\;0\leq \tilde{P}\left(k\right)\leq 1.$$

Thus, the presented function W(j,k) is the joint probability distribution of two random variables j,k satisfying the relation

$$\sum_{j=1}^{2}\sum_{k=1}^{3}W\left(j,k\right)=1.$$

This distribution W(j,k) completely determines the state of spin-1/2 particle for arbitrary probability distributions $\tilde{P}\left(k\right)$.

Now, we consider an explicit generic form of the marginal probability distribution $W_{m}\left(j\right)=\sum_{k=1}^{3}W\left(j,k\right)$; it reads

$$\begin{array}{ccc} & & W_{m}\left(1\right)=p_{1}\tilde{P}\left(1\right)+p_{2}\tilde{P}\left(2\right)+p_{3}\tilde{P}\left(3\right),\\ & & W_{m}\left(2\right)=\left(1-p_{1}\right)\tilde{P}\left(1\right)+\left(1-p_{2}\right)\tilde{P}\left(2\right)+\left(1-p_{3}\right)\tilde{P}\left(3\right). \end{array}$$

A partial case of this generic situation is described if the probability distribution $\tilde{P}\left(k\right)$ is given by equalities $\tilde{P}\left(1\right)=\tilde{P}\left(2\right)=\tilde{P}\left(3\right)=1/3$. One can check that the marginal probability distribution $W_{m}\left(j\right)$ has the form

$$W_{m}\left(1\right)=\frac{1}{3}\left(p_{1}+p_{2}+p_{3}\right),\;W_{m}\left(2\right)=1-\frac{1}{3}\left(p_{1}+p_{2}+p_{3}\right).$$

One can mention that the marginal probability distribution ${\tilde{W}}_{m}\left(k\right)=\sum_{j=1}^{2}W\left(j,k\right)$ coincides with the probability distribution $\tilde{P}\left(k\right)$. The physical interpretation of the probability distribution of two random variable W(j,k) is as follows.

The six available probabilities describe the situation where not only spin projections fluctuate but also directions of *x*, *y*, and *z* axes are random variables since they fluctuate, which is experimentally possible, and this must be taken into account.

The probability representation of qubit states can be illustrated by the geometrical picture constructed as squares named Malevich’s squares [39]. These squares are constructed by means of two triangles. The first triangle is an equilateral triangle with a side length of $\sqrt{2}$. The other triangle is an inscribed equilateral triangle with a side length of 1. The vertices of the inscribed equilateral triangle are located on the sides of the equilateral triangle with the side length of $\sqrt{2}$. It can be shown that the distance from the vertex of the inscribed triangle to the vertex of the large triangle is equal to *x*,

$$x=\frac{\sqrt{2}}{2}\left(1-\frac{1}{\sqrt{3}}\right),$$

and the distance from the second vertex of the large triangle is equal to $\frac{\sqrt{2}}{2}\left(1+\frac{1}{\sqrt{3}}\right).$ The area of three squares with sides equal to 1 constructed, using the equilateral triangle with the sides of this length, is equal to 3. This picture is related to probabilities $p_{1},\;p_{2},\;and,p_{3}$ [39]. This area is the maximum possible area corresponding to the spin-state description by these probabilities $p_{1},\;p_{2},and\;p_{3}$. The spin state is a quantum system, and the analogous Malevich’s squares can be obtained for a classical system of three coins in the game of the coin flipping. In this game, we also have probabilities $p_{1},\;p_{2},and\;p_{3}$ corresponding to the results of the game, if the coins are not ideal, which means that the probabilities $p_{1},\;p_{2},and\;p_{3}$ can take any values from zero to one, and they do not satisfy inequality (6). The triangle picture of this game corresponds to the extremum distances of the vertex of the inscribed triangle, which are equal either to zero or $\sqrt{2}$. This means that in this classical system of three coins, the inscribed triangle can coincide with the large triangle. In addition, this means that the maximum area of three Malevich’s squares constructed, using the sides of the inscribed triangle, is equal to six. Thus, the qubit state, being a quantum system described by three dichotomic probability distributions, and three classical coins, which are also described by three dichotomic distributions, can be distinguished. In fact, the maximum area of the classical system Malevich’s squares is twice as large as the area of such squares for a quantum qubit state.

## 3. The Superposition Principle of Qubit States in the Probability Representation

For pure orthogonal quantum states, the superposition principle for the existing spin states $|\psi_{1}\rangle$ and $|\psi_{2}\rangle$ means that the state vector, given as a linear combination

(8) $$|\psi \rangle =c_{1}|\psi_{1}\rangle +c_{2}|\psi_{2}\rangle$$

of the normalized vectors with complex numbers $c_{1}$ and $c_{2}$ satisfying the condition $\langle \psi |\psi \rangle =|c_{1}{|}^{2}+{\left|c_{2}\right|}^{2}=1$, describes the existing spin state. In [35], the superposition principle was expressed in terms of the density operators ${\hat{\rho }}_{1}=|\psi_{1}\rangle \langle \psi_{1}|$ and ${\hat{\rho }}_{2}=|\psi_{2}\rangle \langle \psi_{2}|$, using the additional pure state density operator ${\hat{\rho }}_{0}=|\psi_{0}\rangle \langle \psi_{0}|$. For orthogonal vectors with ${\hat{\rho }}_{1}{\hat{\rho }}_{2}=0$, the relation of density matrices $\rho_{1},\;\rho_{2},\rho_{0}$, and ρ were expressed as

(9) $$\rho_{\psi }=P_{1}\rho_{1}+P_{2}\rho_{2}+\sqrt{P_{1}P_{2}}\;\frac{\rho_{1}\rho_{0}\rho_{2}+\rho_{2}\rho_{0}\rho_{1}}{\sqrt{\mathrm{Tr}\left(\rho_{1}\rho_{0}\rho_{2}\rho_{0}\right)}},$$

where $P_{1}$ and $P_{2}$ are probabilities, $P_{1}+P_{2}=1$.

We consider an example of the superposition of two pure orthogonal states of the form

(10) $$|\psi_{1}\rangle =\left(\begin{array}{c} 1\\ 0 \end{array}\right),\;|\psi_{2}\rangle =\left(\begin{array}{c} 0\\ 1 \end{array}\right),$$

such that the superposed pure-state vector reads

(11) $$|\psi \rangle =c_{1}\left(\begin{array}{c} 1\\ 0 \end{array}\right)+c_{2}\left(\begin{array}{c} 0\\ 1 \end{array}\right).$$

It describes the density matrix of the state (11) if we use (9). We introduce the notation for the pure-state density matrix using the pure-state normalized vector

(12) $$|\psi_{0}\rangle =\left(\begin{array}{c} c_{1}\\ c_{2} \end{array}\right),$$

which determines the density matrix $\rho_{0}=|\psi_{0}\rangle \langle \psi_{0}|$ as

(13) $$\rho_{0}=\left(\begin{array}{cc} |c_{1}{|}^{2} & c_{1}c_{2}^{*}\\ c_{2}c_{1}^{*} & |c_{2}{|}^{2} \end{array}\right).$$

Applying relation (9), one can see that the density matrix of the superposed state $\rho_{\psi }=\left|\psi \rangle \langle \psi \right|$, which in our example, is equal to $\rho_{0}$ (13) and is given by (9), because the probabilities $P_{1}$ and $P_{2}$ are chosen according to the Born rule

(14) $$P_{1}=\mathrm{Tr}\left(\rho_{0}\rho_{1}\right),\;P_{2}=\mathrm{Tr}\left(\rho_{0}\rho_{2}\right)$$

and

(15) $$\left[\mathrm{Tr}\left(\rho_{1}\rho_{0}\rho_{2}\rho_{0}\right)\right]=P_{1}P_{2}.$$

The obtained relationships such as (9) can be presented in the form of a nonlinear addition rule of probability distributions describing the density matrices $\rho_{\psi }$, $\rho_{1}$, $\rho_{2}$, and $\rho_{0}$ in (9).

## 4. The Probability Representation of Two Spin States

In this section, we consider the states of two spins 1/2 (two qubits) with the density matrix of a ququart state

(16) $$\rho =\left(\begin{array}{cccc} \rho_{11} & \rho_{12} & \rho_{13} & \rho_{14}\\ \rho_{21} & \rho_{22} & \rho_{23} & \rho_{24}\\ \rho_{31} & \rho_{32} & \rho_{33} & \rho_{34}\\ \rho_{41} & \rho_{42} & \rho_{43} & \rho_{44} \end{array}\right).$$

We construct the probability representation, using the Born rule [36,37]. For this, we consider the density matrices of the pure states with the state vectors and corresponding density matrices

(17) $$|z↑,z↑\rangle =\left(\begin{array}{c} 1\\ 0 \end{array}\right)⊗\left(\begin{array}{c} 1\\ 0 \end{array}\right)=\left(\begin{array}{c} 1\\ 0\\ 0\\ 0 \end{array}\right).$$

Here, the used notation means that in this state, the first spin has the spin projection +1/2 on the *z* axis, and the second spin has the projection +1/2 on the *z* axis. This means that the obtained vector is the eigenvector of the 4×4 matrices $S_{z,1},\;S_{z,2}$, which read $S_{z,1}=\frac{1}{2}\sigma_{z}⊗1$ and $S_{z,2}=\frac{1}{2}1⊗\sigma_{z}$ such that $S_{z,1}|z↑,z↑\rangle =\frac{1}{2}|z↑,z↑\rangle$ and $S_{z,2}|z↑,z↑\rangle =\frac{1}{2}|z↑,z↑\rangle$. In addition, $\sigma_{z}$ is the standard Pauli matrix (1) and 1 is the 2×2 identity matrix. The density matrix of this pure state $\rho_{z↑,z↑}=\left|z↑,z↑\rangle \langle z↑,z↑\right|$ has the form

(18) $$\rho_{z↑,z↑}=\left(\begin{array}{cccc} 1 & 0 & 0 & 0\\ 0 & 0 & 0 & 0\\ 0 & 0 & 0 & 0\\ 0 & 0 & 0 & 0 \end{array}\right).$$

The notation describes the state where the first index z↑ means that the spin projection of the first spin along the *z* axis is equal to +1/2, and the second index z↑ means that the spin projection of the second spin along the *z* axis is equal to +1/2. Analogous notation is used for all other vectors, which we introduce below. For example, the vector |z↑,z↓〉 of the form

(19) $$|z↑,z↓\rangle =\left(\begin{array}{c} 1\\ 0 \end{array}\right)⊗\left(\begin{array}{c} 0\\ 1 \end{array}\right)=\left(\begin{array}{c} 0\\ 1\\ 0\\ 0 \end{array}\right)$$

describes the state, where the first spin has the projection equal to +1/2 along the *z* axis, and the second spin has the spin projection −1/2 along the *z* axis. The density matrix of this pure state reads

(20) $$\rho_{z↑,z↓}=\left(\begin{array}{cccc} 0 & 0 & 0 & 0\\ 0 & 1 & 0 & 0\\ 0 & 0 & 0 & 0\\ 0 & 0 & 0 & 0 \end{array}\right).$$

We present below all other analogous eigenvectors, using the introduced notation and corresponding density matrices for all the states, with the spin projections ±1/2 on all the *x*, *y*, and *z* axes. This means that we construct the eigenvectors of the spin projection matrices $S_{x,1}=\frac{1}{2}\sigma_{x}⊗1,$ $S_{x,2}=\frac{1}{2}1⊗\sigma_{x}$, $S_{y,1}=\frac{1}{2}\sigma_{y}⊗1$, and $S_{y,2}=\frac{1}{2}1⊗\sigma_{y}$. The matrices $\sigma_{x},\;\sigma_{y},\;$ and $\sigma_{z}$ are standard Pauli matrices (1). All the eigenvectors and corresponding density matrices are obtained in Appendix A. Calculating the traces of the product of the density matrix (16) with matrices given in Appendix A (18), (20), (A1)–(A22), we obtain the probabilities determining the two qubit states in the probability representation of quantum mechanics. The notation of these probabilities corresponds to the notation of the density matrices. For example, the probability $p_{z↑,z↑}$ due to the Born rule reads $p_{z↑,z↑}=\mathrm{Tr}\left(\rho \rho_{z↑,z↑}\right)$. This relation explicitly is

$$p_{z↑,z↑}=\mathrm{Tr}\left[\left(\begin{array}{cccc} \rho_{11} & \rho_{12} & \rho_{13} & \rho_{14}\\ \rho_{21} & \rho_{22} & \rho_{23} & \rho_{24}\\ \rho_{31} & \rho_{32} & \rho_{33} & \rho_{34}\\ \rho_{41} & \rho_{42} & \rho_{43} & \rho_{44} \end{array}\right)\left(\begin{array}{cccc} 1 & 0 & 0 & 0\\ 0 & 0 & 0 & 0\\ 0 & 0 & 0 & 0\\ 0 & 0 & 0 & 0 \end{array}\right)\right].$$

This means that

(21) $$p_{z↑,z↑}=\rho_{11}.$$

Using the analogous relations presented in Appendix A, we give explicitly the matrix elements of the density matrix expressed in terms of the probabilities $p_{z↑,z↑}$, $p_{z↑,z↓}$, $p_{z↓,z↑},$$p_{z↓,z↓}$, $p_{x↑,x↑}$, $p_{x↑,x↓}$, $p_{x↓,x↑}$, $p_{x↓,↓}$, $p_{y↑,y↑}$, $p_{y↑,y↓}$, $p_{y↓,y↑}$, $p_{y↓,y↓}$, $p_{z↑,x↑}$, $p_{x↑,z↑}$, $p_{x↑,z↓}$, $p_{z↓,x↑}$, $p_{z↑,x↓}$, $p_{x↓,z↑}$, $p_{x↓,z↓}$, $p_{z↓,x↓}$, $p_{z↑,y↑}$, $p_{y↑,z↑}$, $p_{y↑,z↓}$, $p_{z↓,y↑}$, $p_{z↑,y↓}$, $p_{y↓,z↑}$, $p_{y↓,z↓}$, $p_{z↓,y↓}$, $p_{y↑,x↑}$, $p_{x↑,y↑}$, $p_{x↑,y↓}$, $p_{y↓,x↑}$, $p_{y↑,x↓}$, $p_{x↓,y↑}$, $p_{x↓,y↓}$, $p_{y↓,x↓}$ as follows:

(22) $$\begin{array}{ccc} & & \rho_{11}=p_{z↑,z↑},\;\rho_{22}=p_{z↑,z↓},\;\rho_{33}=p_{z↓,z↑},\;\rho_{44}=p_{z↓,z↓},\\ & & \rho_{12}=\rho_{21}^{*}=p_{z↑,x↑}-\frac{1}{2}\left(p_{z↑,z↑}+p_{z↑,z↓}\right)+i\left[\frac{1}{2}\left(p_{z↑,z↑}+p_{z↑,z↓}\right)-p_{z↑,y↑}\right],\\ & & \rho_{13}=\rho_{31}^{*}=p_{x↑,z↑}-\frac{1}{2}\left(p_{z↑,z↑}+p_{z↓,z↑}\right)+i\left[\frac{1}{2}\left(p_{z↑,z↑}+p_{z↑,z↓}\right)-p_{y↑,z↑}\right],\\ & & \rho_{14}=\rho_{41}^{*}=\frac{1}{2}\left(p_{x↑,x↑}+p_{x↓,x↓}-p_{y↑,y↑}-p_{y↓,y↓}\right)\\ & & +\frac{i}{2}\left(p_{y↑,x↓}+p_{y↓,x↑}+p_{x↑,y↓}+p_{x↓,y↑}-1\right),\\ & & \rho_{23}=\rho_{32}^{*}=\frac{1}{2}\left(p_{x↑,x↑}+p_{x↓,x↓}-p_{y↑,y↓}-p_{y↓,y↑}\right)\\ & & +\frac{i}{2}\left(p_{y↑,x↓}+p_{y↓,x↑}-p_{x↑,y↓}-p_{x↓,y↑}\right),\\ & & \rho_{24}=\rho_{42}^{*}=\frac{1}{2}\left(p_{z↑,z↓}+p_{z↓,z↓}\right)-p_{x↓,z↓}+i\left[\frac{1}{2}\left(p_{z↑,z↓}+p_{z↓,z↓}\right)-p_{y↑,z↓}\right],\\ & & \rho_{34}=\rho_{43}^{*}=\frac{1}{2}\left(p_{z↓,z↑}+p_{z↓,z↓}\right)-p_{z↓,x↓}+i\left[\frac{1}{2}\left(p_{z↓,z↑}+p_{z↓,z↓}\right)-p_{z↓,y↑}\right]. \end{array}$$

These formulae provide the possibility to obtain the density matrix of two qubit states by measuring the above probabilities. We point out that for an arbitrary ququart state with the density matrix (16), there exists the formal probability representation described by (22); for example, it exists for a spin-3/2 system, which has no subsystems.

## 5. Bell States in the Probability Representation

In this section, we consider the example of entangled states of two qubits in the probability representation; it is the Bell state which is a superposition of two pure states $\frac{1}{\sqrt{2}}(|z↑,z↑\rangle +\left|z↓,z↓\rangle \right)$. The density matrix of this state reads

(23) $$\rho_{B}=\frac{1}{2}\left(\begin{array}{cccc} 1 & 0 & 0 & 1\\ 0 & 0 & 0 & 0\\ 0 & 0 & 0 & 0\\ 1 & 0 & 0 & 1 \end{array}\right).$$

This state is entangled, and we can check this using the Peres–Horodecki criterion [40,41]. Making the partial transposition operation with the Bell matrix, which has only non-negative eigenvalues, we obtain the matrix of the form

(24) $$\rho_{B}^{ppt}=\frac{1}{2}\left(\begin{array}{cccc} 1 & 0 & 0 & 0\\ 0 & 0 & 1 & 0\\ 0 & 1 & 0 & 0\\ 0 & 0 & 0 & 1 \end{array}\right).$$

One can check that one of the eigenvalues of this matrix is equal to −1/2. This means that the Bell matrix is not separable due to the mentioned criterion [40,41]. In addition, one can obtain the density matrices of the first spin and the second spin using the rule, which is known for an arbitrary 4×4 matrix presented in the block form,

(25) $$\rho =\left(\begin{array}{cc} A & B\\ C & D \end{array}\right),$$

where A,B,C, and D are 2×2 matrices [42]. According to this rule, the first spin density matrix is expressed as follows:

(26) $$\rho^{\left(1\right)}=\left(\begin{array}{cc} \mathrm{Tr}A & \mathrm{Tr}B\\ \mathrm{Tr}C & \mathrm{Tr}D \end{array}\right),$$

and the density matrix of the second spin reads

(27) $$\rho^{\left(2\right)}=A+D.$$

Considering Equation (22) for a general matrix of the form (25) and calculating the matrices $\rho^{\left(1\right)}$ (26) and $\rho^{\left(2\right)}$ (27) in this form, we obtain their matrix elements; they read

(28) $$\begin{array}{ccc} & & \rho_{11}^{\left(1\right)}=p_{z↑,z↑}+p_{z↑,z↓},\;\rho_{11}^{\left(2\right)}=p_{z↑,z↑}+p_{z↓,z↑},\\ & & \rho_{12}^{\left(1\right)}=p_{x↑,z↑}-\frac{1}{2}\left(p_{z↑,z↑}+p_{z↓,z↑}\right)+\frac{1}{2}\left(p_{z↑,z↓}+p_{z↓,z↓}\right)-p_{x↓,z↓}\\ & & +i\left[\frac{1}{2}\left(p_{z↑,z↑}+p_{z↓,z↑}\right)-p_{y↑,z↑}+\frac{1}{2}\left(p_{z↑,z↓}+p_{z↓,z↓}\right)-p_{y↑,z↓}\right],\\ & & \rho_{12}^{\left(2\right)}=p_{z↑,x↑}-\frac{1}{2}\left(p_{z↑,z↑}+p_{z↑,z↓}\right)+\frac{1}{2}\left(p_{z↓,z↑}+p_{z↓,z↓}\right)-p_{z↓,x↓}\\ & & +i\left[\frac{1}{2}\left(p_{z↑,z↑}+p_{z↑,z↓}\right)-p_{z↑,y↑}+\frac{1}{2}\left(p_{z↓,z↑}+p_{z↓,z↓}\right)-p_{z↓,y↑}\right]. \end{array}$$

For the matrix elements, we use the equalities $\rho_{12}^{\left(1\right)}={\left(\rho_{21}^{\left(1\right)}\right)}^{*}$, $\rho_{12}^{\left(2\right)}={\left(\rho_{21}^{\left(2\right)}\right)}^{*}$, $\rho_{22}^{\left(1\right)}=1-\rho_{11}^{\left(1\right)}$, $\rho_{22}^{\left(2\right)}=1-\rho_{11}^{\left(2\right)}$. Thus, we expressed the probabilities of the qubit states determining the 2×2 matrices of the first and second spins in terms of the probabilities determining the 4×4 density matrix in terms of the probabilities of the spin projection of both spins on the *x*, *y*, and *z* axes. These relations are valid for both separable and entangled states. According to the Silvester criterion, all the probabilities satisfy inequalities for minors of the 2×2 and 4×4 density matrices. One can check that the probabilities determining the entangled Bell states will satisfy all these discussed equalities and inequalities. Applying the rules (26) and (27) to the density matrix of the entangled Bell state (23), we see that the density matrix of the first and second spins in the Bell states is

(29) $$\rho^{\left(1\right)}=\rho^{\left(2\right)}=\left(\begin{array}{cc} 1/2 & 0\\ 0 & 1/2 \end{array}\right).$$

According to (5), we have $p_{1}^{\left(1\right)}=p_{2}^{\left(1\right)}=p_{3}^{\left(1\right)}=P_{1}^{\left(2\right)}=P_{2}^{\left(2\right)}=P_{3}^{\left(2\right)}=1/2$, where the numbers $p_{k}^{\left(1\right)},\;P_{k}^{\left(2\right)}$(k=1,2,3) are probabilities determining the spin-1/2 states according to (5). In these states, both spins have equal probabilities, namely, 1/2 to be directed along the x,y, and z axes.

Now, we present the probability representation of the Bell states as the expression of the 4×4 density matrix, using the general formula (22)

(30) $$\begin{array}{ccc} & & p_{x↑,x↑}=p_{x↓,x↓}=p_{z↑,z↑}=p_{z↓,z↓}=p_{y↑,y↓}=p_{y↓,y↑}=\frac{1}{2},\\ & & p_{x↑,x↓}=p_{x↓,x↑}=p_{z↑,z↓}=p_{z↓,z↑}=p_{y↑,y↑}=p_{y↓,y↓}=0,\\ & & p_{z↑,x↑}=p_{x↑,z↑}=p_{x↓,z↓}=p_{z↓,x↓}=p_{z↑,x↓}=p_{x↓,z↑}=p_{y↑,x↓}=p_{x↓,y↑}=p_{x↑,y↓}\\ & & =p_{y↓,x↑}=p_{z↑,y↑}=p_{y↑,z↑}=p_{y↓,z↓}=p_{z↓,y↓}=p_{y↑,z↓}=p_{z↓,y↑}=\frac{1}{4}. \end{array}$$

The matrices $\rho^{\left(1\right)}$ and $\rho^{\left(2\right)}$ with matrix elements (30) satisfy inequality (6).

## 6. Linear Entropy of Entangled Qubit States

Let us consider the linear entropy according to the following formula [43]:

(31) $$H=1-\mathrm{Tr}{\left(\rho^{\left(1\right)}\right)}^{2}.$$

If the separable state of two qubits with the state-vector |ψ〉 is of the form $\left|\psi \rangle =\right|\psi_{1}\rangle |\psi_{2}\rangle$, where $|\psi_{1}\rangle$ and $|\psi_{2}\rangle$ are the two-dimensional state vectors, the density 2×2 matrices are $\rho^{\left(1\right)}=|\psi_{1}\rangle \langle \psi_{1}|$ and $\rho^{\left(2\right)}=|\psi_{2}\rangle \langle \psi_{2}|$; in this case, ${\left(\rho^{\left(1\right)}\right)}^{2}=\rho^{\left(1\right)}$, and this means that the linear entropy (31) is equal to zero.

If the density matrix of the two qubit pure state |ψ〉〈ψ| is entangled, we have $|\psi \rangle =N(|\phi_{1}\rangle +|\psi_{1}\rangle )$ and $\langle \psi |=N(\langle \phi_{1}|+\langle \psi_{1}|)$. The linear entropy for such states is not equal to zero, because $\mathrm{Tr}{\left(\rho^{\left(1\right)}\right)}^{2}<1$.

Let us calculate $\mathrm{Tr}{\left(\rho^{\left(1\right)}\right)}^{2}$ in the probability representation of quantum states

(32) $$\mathrm{Tr}{\left(\rho^{\left(1\right)}\right)}^{2}={\left(p_{3}^{\left(1\right)}\right)}^{2}+2{\left|P\right|}^{2}+{\left(\left(1-p_{3}^{\left(1\right)}\right)\right)}^{2},$$

where $P=p_{1}^{\left(1\right)}-1/2+i\left(p_{2}^{\left(1\right)}-1/2\right)$.

For the pure state, $\mathrm{Tr}{\left(\rho^{\left(1\right)}\right)}^{2}=1$. If we consider *H* for arbitrary entangled two-qubit states, it is expressed through

$$\mathrm{Tr}{\left(\rho^{\left(1\right)}\right)}^{2}={\left(\rho_{11}^{\left(1\right)}\right)}^{2}+2{\left|\rho_{12}\right|}^{2}+\rho_{22}^{\left(2\right)},$$

and its explicit form reads

(33) $$\begin{array}{ccc} & & H=1-p_{z↑,z↑}-2\left\{\left[p_{z↑,x↑}-\frac{1}{2}\left(p_{z↑,z↑}+p_{z↑,a↓}\right)\right]+{\left[\frac{1}{2}{\left(p_{z↑,z↑}+p_{z↑,z↓}\right)}^{2}-p_{z↑,y↑}\right]}^{2}\right\}\\ & & -{\left(1-p_{z↑,z↑}\right)}^{2}. \end{array}$$

This means that the linear entropy is not equal to zero for the entangled states of two qubits. One can check that for Bell states, the linear entropy is equal to 1/2 (29). Thus, we expressed the linear entropy in terms of the probabilities for arbitrary two-qubit states.

## 7. The Entanglement of Two-Mode Oscillator States in the Probability Representation of Quantum Mechanics

Now, we consider the two-oscillator Gaussian states in the tomographic probability representation. To obtain the tomogram of such states, we recall that for one oscillator state, one should use the dequantizer operator $\hat{U}\left(X,\mu ,\nu \right)=\delta \left(X\hat{1}-\mu \hat{q}-\nu \hat{p}\right),$ where $\hat{q}$ and $\hat{p}$ are the position and momentum operators in the position representation [44,45]. One can check that the matrix elements of this operator read

(34) $$\langle x|\delta \left(X\hat{1}-\mu \hat{q}-\nu \hat{p}\right)|x^{\prime }\rangle =\frac{1}{2\pi |\nu |}\exp\left(-\frac{i\mu }{2\nu }x^{2}+\frac{i\mu }{2\nu }{x^{\prime }}^{2}+\frac{i}{\nu }Xx-\frac{i}{\nu }Xx^{\prime }\right).$$

This formula can be presented in the other form

(35) $$\langle x|\delta \left(X\hat{1}-\mu \hat{q}-\nu \hat{p}\right)|x^{\prime }\rangle =\frac{1}{2\pi |\nu |}\exp\left[\frac{i}{\nu }\left(x-x^{\prime }\right)\left(X-\frac{\mu }{2}\left(x+x^{\prime }\right)\right)\right].$$

The tomogram w(X|μ,ν) of a one-mode state with a density operator $\hat{\rho }$ (called the tomographic symbol of the operator $\hat{\rho }$) is $w\left(X|\mu ,\nu \right)=\mathrm{Tr}\rho \hat{U}\left(X,\mu ,\nu \right)$. The density operator of the one oscillator state can be reconstructed by means of the quantizer operator $\hat{D}\left(X,\mu ,\nu \right)=\frac{1}{2\pi }\exp\left(X\hat{1}-\nu \hat{q}-\nu \hat{p}\right)$ such that

(36) $$\hat{\rho }=\int w\left(X|\mu ,\nu \right)\hat{D}\left(X,\mu ,\nu \right)dX\;d\mu \;d\nu .$$

The matrix elements of the quantizer operator in the position representation have the form

(37) $$\frac{1}{2\pi }\langle x|\exp\left(X\hat{1}-\mu \hat{q}-\nu \hat{p}\right)|x^{\prime }\rangle =\frac{1}{2\pi }\exp\left[i\left(X-\frac{\mu }{2}\left(x+x^{\prime }\right)\right)\right]\delta \left(x-x^{\prime }-\nu \right).$$

The tomogram of the state with the state vector |ψ〉 and wave function ψ(z) is

(38) $$\mathrm{Tr}\left(|\psi \rangle \langle \psi |\delta \left(X\hat{1}-\nu \hat{q}-\nu \hat{p}\right)\right)=\frac{1}{2\pi |\nu |}{\left|\int dz\;\psi \left(z\right)\exp\left(\frac{i\mu }{2\nu }z^{2}-\frac{i}{\nu }Xz\right)\right|}^{2}$$

The tomographic symbol of the operators |ϕ〉〈ψ| determined by state vectors |ϕ〉 and |ψ〉 of a one-mode oscillator is

(39) $$\begin{array}{ccc} & & \mathrm{Tr}\left(|\phi \rangle \langle \psi |\delta \left(X\hat{1}-\nu \hat{q}-\nu \hat{p}\right)\right)=\frac{1}{2\pi |\nu |}\int dx\;\phi^{*}\left(x\right)\exp\left(-\frac{i\mu }{2\nu }x^{2}+\frac{i}{\nu }Xx\right)\\ & & \times \int dx^{\prime }\;\psi \left(x^{\prime }\right)\exp\left(\frac{i\mu }{2\nu }{x^{\prime }}^{2}-\frac{i}{\nu }Xx^{\prime }\right) \end{array}$$

If |ϕ〉 is the ground state of oscillator $|\phi_{0}\rangle$ and |ψ〉 is the coherent state $|\psi_{\alpha }\rangle$, then (39) is expressed as an integral,

(40) $$\begin{array}{ccc} & & \mathrm{Tr}\left(|\phi_{0}\rangle \langle \psi_{\alpha }|\delta \left(X\hat{1}-\nu \hat{q}-\nu \hat{p}\right)\right)=\frac{1}{2\pi |\nu |}\int \frac{dx}{\pi^{1/4}}\;\exp\left(-\frac{x^{2}}{2}\right)\exp\left(-\frac{i\mu }{2\nu }x^{2}+\frac{i}{\nu }Xx\right)\\ & & \times \int \frac{dx^{\prime }}{\pi^{1/4}}\;\exp\left(-\frac{{x^{\prime }}^{2}}{2}-\frac{{\left|\alpha \right|}^{2}}{2}-\frac{\alpha^{2}}{2}+\sqrt{2}\alpha x^{\prime }\right)\exp\left(\frac{i\mu }{2\nu }{x^{\prime }}^{2}-\frac{i}{\nu }Xx^{\prime }\right). \end{array}$$

Calculating the Gaussian integral, we arrive at

(41) $$\begin{array}{ccc} & & \mathrm{Tr}\left(|\phi_{0}\rangle \langle \psi_{\alpha }|\delta \left(X\hat{1}-\nu \hat{q}-\nu \hat{p}\right)\right)\\ & & =\frac{1}{\sqrt{\pi (\mu^{2}+\nu^{2})}}\exp\left[-\frac{{\left|\alpha \right|}^{2}}{2}+\frac{\alpha^{2}\left(\nu +i\mu \right)}{2(\nu -i\mu )}-\frac{i\sqrt{2}\alpha X}{\nu -i\mu }-\frac{X^{2}}{\mu^{2}+\nu^{2}}\right]. \end{array}$$

Let us consider the superposition state

(42) $$|\Phi \rangle =N\left(|\phi \rangle +|\psi \rangle \right),$$

where |ϕ〉 and |ψ〉 are any normalized states, i.e., 〈ϕ|ϕ〉=1 and 〈ψ|ψ〉=1. Then, the state |Φ〉 is normalized, if

(43) $$N^{2}=\frac{1}{2+\langle \psi |\phi \rangle +\langle \phi |\psi \rangle }.$$

The tomogram of such a state reads

(44) $$\begin{array}{ccc} & & w\left(X|\mu ,\nu \right)=\mathrm{Tr}\left[\delta \left(X\hat{1}-\mu \hat{q}-\nu \hat{p}\right)\left(|\phi \rangle +|\psi \rangle \right)\left(\langle \phi |+\langle \psi |\right)N^{2}\right]=\\ & & N^{2}\mathrm{Tr}\left[\delta \left(X\hat{1}-\mu \hat{q}-\nu \hat{p}\right)\left(|\phi \rangle \langle \phi |+|\psi \rangle \langle \psi +|\phi \rangle \langle \psi |+|\psi \rangle \langle \phi |\right)\right]=\\ & & N^{2}\left\{\mathrm{Tr}\left[\delta \left(X\hat{1}-\mu \hat{q}-\nu \hat{p}\right)\left|\phi \rangle \langle \phi \right|\right]+\mathrm{Tr}\left[\delta \left(X\hat{1}-\mu \hat{q}-\nu \hat{p}\right)|\psi \rangle \langle \psi \right]\right.\\ & & \left.+\mathrm{Tr}\left[\delta \left(X\hat{1}-\mu \hat{q}-\nu \hat{p}\right)\left|\phi \rangle \langle \psi \right|\right]+\mathrm{Tr}\left[\delta \left(X\hat{1}-\mu \hat{q}-\nu \hat{p}\right)\left|\psi \rangle \langle \phi \right|\right]\right\} \end{array}$$

Let us consider a two-mode oscillator state with the wave function Φ(x,y). Its tomogram $w(X_{1},X_{2}|\mu_{1},\nu_{1},\mu_{2},\nu_{2})$ is given by

(45) $$w\left(X_{1},X_{2}|\mu_{1},\nu_{1},\mu_{2},\nu_{2}\right)=\mathrm{Tr}\left[|\Phi \rangle \langle \Phi |\delta \left(X_{1}\hat{1}-\mu_{1}{\hat{q}}_{1}-\nu_{1}{\hat{p}}_{1}\right)\delta \left(X_{2}\hat{1}-\mu_{2}{\hat{q}}_{2}-\nu_{2}{\hat{p}}_{2}\right)\right],$$

where |Φ〉 is the state vector corresponding to the wave function Φ(x,y). Formula (45) can be written in the form of integral

(46) $$\begin{array}{ccc} & & w\left(X_{1},X_{2}|\mu_{1},\nu_{1},\mu_{2},\nu_{2}\right)=\frac{1}{4\pi^{2}\left|\nu_{1}\nu_{2}\right|}\int \Phi \left(x,y\right)\Phi^{*}\left(x^{\prime },y^{\prime }\right)\exp\left(-\frac{i\mu_{1}x^{2}}{2\nu_{1}}+\frac{i\mu_{1}x^{\prime 2}}{2\nu_{1}}\right.\\ & & \left.+\frac{iX_{1}x}{\nu_{1}}-\frac{iX_{1}x^{\prime }}{\nu_{1}}-\frac{i\mu_{2}y^{2}}{2\nu_{2}}-\frac{i\mu_{2}y^{\prime 2}}{2\nu_{2}}+\frac{iX_{2}y}{\nu_{2}}-\frac{iX_{2}y^{\prime }}{\nu_{2}}\right)dxdx^{\prime }dydy^{\prime }. \end{array}$$

The state vector under study corresponds to the superposition state of the ground and coherent states of the two-mode oscillator (the Schrödinger cat state)

(47) $$|\Phi \rangle =\frac{1}{\sqrt{2(1+e^{-{\left|\alpha \right|}^{2}})}}(|\phi_{0}\rangle |\psi_{\alpha }\rangle +|\phi_{\alpha }\rangle |\psi_{o}\rangle ),$$

where $|\phi_{0}\rangle$ and $|\psi_{0}\rangle$ are state vectors of ground states of the first and second modes, and $|\phi_{\alpha }\rangle$ and $|\psi_{\alpha }\rangle$ are state vectors of coherent states of the first and second modes. For the density matrix of the superposition state of the two-mode oscillator, we obtain

(48) $$\begin{array}{ccc} & & \left|\Phi \rangle \langle \Phi \right|=N^{2}\left(|\phi_{0}\rangle |\psi_{\alpha }\rangle +|\phi_{\alpha }\rangle |\psi_{0}\rangle \right)\left(\langle \phi_{0}|\langle \psi_{\alpha }|+\langle \phi_{\alpha }\left|\langle \right|\psi_{0}|\right)=N^{2}\left(|\phi_{0}\rangle \langle \phi_{0}\left|⊗\right|\psi_{\alpha }\rangle \langle \psi_{\alpha }|\right.\\ & & +\left.|\phi_{\alpha }\rangle \langle \phi_{\alpha }\left|⊗\right|\psi_{0}\rangle \langle \psi_{0}\left|+\right|\phi_{\alpha }\rangle \langle \phi_{0}\left|⊗\right|\psi_{0}\rangle \langle \psi_{\alpha }\left|+\right|\phi_{0}\rangle \langle \phi_{\alpha }\left|⊗\right|\psi_{\alpha }\rangle \langle \psi_{0}|\right) \end{array}$$

The tomogram of state (45) can be written in the form of the sum of four terms; each of them is the product of the Gaussian integrals,

(49) $$\begin{array}{ccc} & & w(X_{1},X_{2}|\mu_{1},\nu_{1},\mu_{2},\nu_{2})=\\ & & \frac{N^{2}}{2\pi |\nu_{1}\nu_{2}|}\left\{{\left|\int dx\phi_{0}\left(x\right)\exp\left[\frac{i\mu_{1}x^{2}}{2\nu_{1}}-\frac{iX_{1}x}{\nu_{1}}\right]\right|}^{2}{\left|\int dy\psi_{\alpha }\left(y\right)\exp\left[\frac{i\mu_{2}y^{2}}{2\nu_{2}}-\frac{iX_{2}y}{\nu_{2}}\right]\right|}^{2}\right.\\ & & +{\left|\int dy\psi_{0}\left(y\right)\exp\left[\frac{i\mu_{2}y^{2}}{2\nu_{2}}-\frac{iX_{2}y}{\nu_{2}}\right]\right|}^{2}{\left|\int dx\phi_{\alpha }\left(x\right)\exp\left[\frac{i\mu_{1}x^{2}}{2\nu_{1}}-\frac{iX_{1}x}{\nu_{1}}\right]\right|}^{2}\\ & & +\int dy\phi_{\alpha }\left(x^{\prime }\right)\exp\left[\frac{i\mu_{1}{x^{\prime }}^{2}}{2\nu_{1}}-\frac{iX_{1}x^{\prime }}{\nu_{1}}\right]\int dx\phi_{0}^{*}\left(x\right)\exp\left[\frac{-i\mu_{1}x^{2}}{2\nu_{1}}+\frac{iX_{1}x}{\nu_{1}}\right]\\ & & \left.\times \int dy^{\prime }\psi_{0}\left(y^{\prime }\right)\exp\left[\frac{i\mu_{2}{y^{\prime }}^{2}}{2\nu_{2}}-\frac{iX_{2}x^{\prime }}{\nu_{2}}\right]\int dy\psi_{\alpha }^{*}\left(y\right)\exp\left[\frac{-i\mu_{2}y^{2}}{2\nu_{2}}+\frac{iX_{2}x}{\nu_{2}}\right]\right\}, \end{array}$$

where the wave functions of ground states read

$$\phi_{0}\left(x\right)=\frac{1}{\pi^{1/4}}\exp\left(-\frac{x^{2}}{2}\right),\;\psi_{0}\left(y\right)=\frac{1}{\pi^{1/4}}\exp\left(-\frac{y^{2}}{2}\right)$$

and the wave functions of coherent states are

$$\phi_{\alpha }\left(x\right)=\frac{1}{\pi^{1/4}}\exp\left(-\frac{x^{2}}{2}+\sqrt{2}\alpha x-\frac{{\left|\alpha \right|}^{2}}{2}-\frac{\alpha^{2}}{2}\right),$$

$$\psi_{\alpha }\left(y\right)\frac{1}{\pi^{1/4}}\exp\left(-\frac{y^{2}}{2}+\sqrt{2}\alpha y-\frac{{\left|\alpha \right|}^{2}}{2}-\frac{\alpha^{2}}{2}\right).$$

The explicit form of tomogram (45) is

(50) $$\begin{array}{ccc} & & w(X_{1},X_{2}|\mu_{1},\nu_{1},\mu_{2},\nu_{2})=\\ & & \frac{\exp\left(-{\left|\alpha \right|}^{2}-\frac{\alpha^{2}}{2}-\frac{\alpha^{*2}}{2}\right)}{2\pi \left(1+e^{-{\left|\alpha \right|}^{2}}\right)\sqrt{\left(\mu_{1}^{2}+\nu_{1}^{2}\right)\left(\mu_{2}^{2}+\nu_{2}^{2}\right)}}\exp\left(-\frac{X_{1}^{2}}{\mu_{1}^{2}+\nu_{1}^{2}}\right)\exp\left(-\frac{X_{2}^{2}}{\mu_{2}^{2}+\nu_{2}^{2}}\right)\\ & & \times \left\{\exp\left[\frac{\left(\alpha^{2}+\alpha^{*2}\right)\nu_{2}^{2}}{\mu_{2}^{2}+\nu_{2}^{2}}-\frac{i\sqrt{2}\nu_{2}\left(\alpha -\alpha^{*}\right)X_{2}}{\mu_{2}^{2}+\nu_{2}^{2}}+\frac{i\left(\alpha^{2}-\alpha^{*2}\right)\mu_{2}\nu_{2}}{\mu_{2}^{2}+\nu_{2}^{2}}+\frac{\sqrt{2}X_{2}\mu_{2}\left(\alpha +\alpha^{*}\right)}{\mu_{2}^{2}+\nu_{2}^{2}}\right]\right.\\ & & \left.+\exp\left[\frac{\left(\alpha^{2}+\alpha^{*2}\right)\nu_{1}^{2}}{\mu_{1}^{2}+\nu_{1}^{2}}-\frac{i\sqrt{2}\nu_{1}\left(\alpha -\alpha^{*}\right)X_{1}}{\mu_{1}^{2}+\nu_{1}^{2}}+\frac{i\left(\alpha^{2}-\alpha^{*2}\right)\mu_{1}\nu_{1}}{\mu_{1}^{2}+\nu_{1}^{2}}+\frac{\sqrt{2}X_{1}\mu_{1}\left(\alpha +\alpha^{*}\right)}{\mu_{1}^{2}+\nu_{1}^{2}}\right]\right.\\ & & \left.+\exp\left[\frac{\alpha^{2}\nu_{1}^{2}}{\mu_{1}^{2}+\nu_{1}^{2}}+\frac{\alpha^{*2}\nu_{2}^{2}}{\mu_{2}^{2}+\nu_{2}^{2}}+\frac{i\alpha^{2}\mu_{1}\nu_{1}}{\mu_{1}^{2}+\nu_{1}^{2}}-\frac{i\alpha^{*2}\mu_{2}\nu_{2}}{\mu_{2}^{2}+\nu_{2}^{2}}+\frac{\sqrt{2}\alpha \left(\mu_{1}-i\nu_{1}\right)X_{1}}{\mu_{1}^{2}+\nu_{1}^{2}}\right.\right.\\ & & \left.+\frac{\sqrt{2}\alpha^{*2}\left(\mu_{2}+i\nu_{2}\right)X_{2}}{\mu_{2}^{2}+\nu_{2}^{2}}\right]\\ & & +\exp\left[\frac{\alpha^{2}\nu_{2}^{2}}{\mu_{2}^{2}+\nu_{2}^{2}}+\frac{\alpha^{*2}\nu_{1}^{2}}{\mu_{1}^{2}+\nu_{1}^{2}}+\frac{i\alpha^{2}\mu_{2}\nu_{2}}{\mu_{2}^{2}+\nu_{2}^{2}}-\frac{i\alpha^{*2}\mu_{1}\nu_{1}}{\mu_{1}^{2}+\nu_{1}^{2}}+\frac{\sqrt{2}\alpha \left(\mu_{2}-i\nu_{2}\right)X_{2}}{\mu_{2}^{2}+\nu_{2}^{2}}\right.\\ & & \left.\left.+\frac{\sqrt{2}\alpha^{*2}\left(\mu_{1}+i\nu_{1}\right)X_{1}}{\mu_{1}^{2}+\nu_{1}^{2}}\right]\right\}. \end{array}$$

This tomogram of the two-mode oscillator state is the conditional probability distribution of two random variables $X_{1}$ and $X_{2}$ satisfying the relation

(51) $$\int w\left(X_{1},X_{2}|\mu_{1},\nu_{1},\mu_{2},\nu_{2}\right)\;dX_{1}\;dX_{2}=1.$$

In addition,

(52) $$\int w\left(X_{1},X_{2}|\mu_{1},\nu_{1},\mu_{2},\nu_{2}\right)\;dX_{2}=\tilde{w}\left(X_{1}|\mu_{1},\nu_{1}\right),$$

which is the conditional probability distribution determining the state of the first oscillator. Tomogram (46) is the tomogram of an entangled state with the density matrix given by (48), which is the density matrix of the superposition state of the two-mode oscillator.

One can check that the density operator of the first oscillator ${\hat{\rho }}^{\left(1\right)}$ determined by the tomogram $\tilde{w}\left(X_{1}|\mu_{1},\nu_{1}\right)$, in view of (36), satisfies the condition

(53) $$\mathrm{Tr}{\left(\rho^{\left(1\right)}\right)}^{2}=\int \tilde{w}\left(X_{1}|\mu_{1},\nu_{1}\right)\tilde{w}\left(Y_{1}|-\mu_{1},-\nu_{1}\right)\exp\left(i(X_{1}+Y_{1})\right)dX_{1}\;dY_{1}\;d\mu_{1}\;d\;\nu_{1}<1.$$

The linear entropy is $H=1-\mathrm{Tr}{\left({\hat{\rho }}^{\left(1\right)}\right)}^{2}$, and this means that the linear entropy is not equal to zero; i.e., the state (47) is entangled. It is interesting that the usual normal probability distributions are given in the Gaussian forms [46,47]. The properties of probability distributions characterizing different aspects of quantum states are discussed in [48,49]. However, in the case of entangled quantum oscillator states, it appears that the probability distributions, being the sums of Gaussian terms, provide the specific formal probability distributions in classical probability theory related to quantum mechanics and the description of entanglement phenomena.

## 8. Conclusions

To conclude, we formulate the main results of our work.

Using the new representation of quantum system states, namely, the probability representation, where the states are identified with probability distributions, we explicitly construct any density matrix of the spin-1/2 states. The matrix elements of this density matrix are expressed in terms of probability distributions of two spin projections onto directions of the *x*, *y*, and *z* axes in three-dimensional space. The construction is based on the probability representation of the density matrix elements for one qubit states, which are expressed in terms of dichotomic probabilities to have the spin projections onto the *x*, *y*, and *z* axes. The method to find the explicit expressions of the density matrix is based on the Born rule [36,37]. First, we constructed the vectors in the Hilbert space, which are eigenvectors of the spin-projection operators providing the expressions for density matrices of these states and then, applying the Born rule, we found all corresponding probabilities, which are needed for expressing any density matrix elements in terms of the probabilities. We considered, in the probability representation, a particular example of the entangled Bell state. Since the probability distributions determine the associated Shannon entropy, the new entropies of quantum qubit states different from the von Neumann entropy can be introduced and found for the Bell states. The linear entropy of entangled Bell states was studied in the probability representation. The probability representation of the entangled Schrödinger cat states of the two-mode oscillator also was considered on the example of the superposition of the products of the coherent state of the first oscillator and the ground state of the second oscillator. The entanglement of this state was studied within the framework of the symplectic tomographic probability distributions determining the Schrödinger cat states. The obtained probability distribution is expressed in terms of the sum of the Gaussian terms of the form analogous to the form of tomograms of the Schrödinger cat states of free particles [30]. It is worthwhile to add that different aspects of the Bell inequalities, quantum dynamics, and probability and quasiprobability representations and their applications were considered recently in [50,51,52,53,54,55]. The tomographic linear entropy of the Schrödinger cat states is introduced, and the linear entropy characterizing the entanglement of these states is obtained. The obtained results can be generalized for *n*-qubit and qudit states as well as for multimode superpositions of the Gaussian states with different symmetries discussed in [56].

We can address the question: what new knowledge can be obtained using the studied probability representation of quantum states in comparison with using the standard representation of quantum mechanics? The statistical properties of quantum systems can be considered using the language of classical probability theory. Namely, we formulated in the language of the tomograms determining the quantum states, which are the standard probability distribution functions (not state vectors or density operators), the notion of entanglement of the Bell states of two qubits. It is worthwhile to mention that the Shannon entropy associated with the tomographic probabilities can give extra characteristics of the properties of quantum system states, which were not used in the standard representations of quantum mechanics in the literature. In addition, all the known inequalities for classical probability distributions for the Shannon entropy and other entropies (the Tsallis entropy) can be applied in quantum mechanics using the new formalism of probability distributions determining the quantum states. The new aspect of the formalism presented in the paper is a possible application of the tomographic approach to quantum statistics and description of the system in the state of the thermodynamic equilibrium. For systems with Hamiltonian $\hat{H}$ and temperature *T*, these states have density operators $\hat{\rho }={\left(Z(T)\right)}^{-1}\exp\left(-\hat{H}/T\right)$ where $Z\left(T\right)=\mathrm{Tr}\left(\exp\left(-\hat{H}/T\right)\right)$. In the probability representation of quantum states, this operator is mapped onto the tomographic probability distribution describing this state of the system at temperature *T*. We will study the above-mentioned approach to quantum statistics in future publications. 

## Appendix A

In the Appendix, we present calculations of state vectors and density matrices of specific pure states of two spins-1/2 systems used to calculate the probabilities determining the states of two qubits.

The state vector and the density matrix of the state, where the first spin has the projection equal to −1/2 and the second spin has the projection equal to +1/2 on the *z* axis, have the form

(A1) $$|z↓,z↑\rangle =\left(\begin{array}{c} 0\\ 1 \end{array}\right)⊗\left(\begin{array}{c} 1\\ 0 \end{array}\right)=\left(\begin{array}{c} 0\\ 0\\ 1\\ 0 \end{array}\right),\;\rho_{z↓,z↑}=\left(\begin{array}{cccc} 0 & 0 & 0 & 0\\ 0 & 0 & 0 & 0\\ 0 & 0 & 1 & 0\\ 0 & 0 & 0 & 0 \end{array}\right).$$

The state vector and the density matrix of the state, where the first and second spins have the projections on the *z* axis equal to −1/2, read

(A2) $$|z↓,z↓\rangle =\left(\begin{array}{c} 0\\ 1 \end{array}\right)⊗\left(\begin{array}{c} 0\\ 1 \end{array}\right)=\left(\begin{array}{c} 0\\ 0\\ 0\\ 1 \end{array}\right),\;\rho_{z↓,z↓}=\left(\begin{array}{cccc} 0 & 0 & 0 & 0\\ 0 & 0 & 0 & 0\\ 0 & 0 & 0 & 0\\ 0 & 0 & 0 & 1 \end{array}\right).$$

The state vector and the density matrix of the state, where the first and second spins have the projections on the *x* axis equal to +1/2, are

(A3) $$|x↑,x↑\rangle =\left(\begin{array}{c} 1/\sqrt{2}\\ 1/2\sqrt{2} \end{array}\right)⊗\left(\begin{array}{c} 1\sqrt{2}\\ 1\sqrt{2} \end{array}\right)=\left(\begin{array}{c} 1/2\\ 1/2\\ 1/2\\ 1/2 \end{array}\right),\;\rho_{x↑,x↑}=\left(\begin{array}{cccc} 1/4 & 1/4 & 1/4 & 1/4\\ 1/4 & 1/4 & 1/4 & 1/4\\ 1/4 & 1/4 & 1/4 & 1/4\\ 1/4 & 1/4 & 1/4 & 1/4 \end{array}\right).$$

The state vector and the density matrix of the state, where the first spin has the projection equal to +1/2 and the second spin has the projection equal to −1/2 on *x* axis, have the form

(A4) $$|x↑,x↓\rangle =\left(\begin{array}{c} 1/2\\ -1/2\\ 1/2\\ -1/2 \end{array}\right),\;\rho_{x↑,x↓}=\left(\begin{array}{cccc} 1/4 & -1/4 & 1/4 & -1/4\\ -1/4 & 1/4 & -1/4 & 1/4\\ 1/4 & -1/4 & 1/4 & -1/4\\ -1/4 & 1/4 & -1/4 & 1/4 \end{array}\right).$$

The state vector and the density matrix of the state, where the first spin has the projection equal to −1/2 and the second spin has the projection equal to +1/2 on the *x* axis, have the form

(A5) $$|x↓,x↑\rangle =\left(\begin{array}{c} 1/2\\ 1/2\\ -1/2\\ -1/2 \end{array}\right),\;\rho_{x↓,x↑}=\left(\begin{array}{cccc} 1/4 & 1/4 & -1/4 & -1/4\\ 1/4 & 1/4 & -1/4 & -1/4\\ -1/4 & -1/4 & 1/4 & 1/4\\ -1/4 & -1/4 & 1/4 & 1/4 \end{array}\right).$$

The state vector and the density matrix of the state, where the first and second spins have the projections on the *x* axis equal to −1/2, are

(A6) $$|x↓,x↓\rangle =\left(\begin{array}{c} 1/2\\ -1/2\\ -1/2\\ 1/2 \end{array}\right),\;\rho_{x↓,x↓}=\left(\begin{array}{cccc} 1/4 & -1/4 & -1/4 & 1/4\\ -1/4 & 1/4 & 1/4 & -1/4\\ -1/4 & 1/4 & 1/4 & -1/4\\ 1/4 & -1/4 & -1/4 & 1/4 \end{array}\right).$$

The state vector and the density matrix of the state, where the first and second spins have the projections on *y* axis equal to +1/2, are

(A7) $$|y↑,y↑\rangle =\left(\begin{array}{c} 1/2\\ i/2\\ i/2\\ -1/2 \end{array}\right),\;\rho_{y↑,y↑}=\left(\begin{array}{cccc} 1/4 & -i/4 & -i/4 & -1/4\\ i/4 & 1/4 & 1/4 & -i/4\\ i/4 & 1/4 & 1/4 & -i/4\\ -1/4 & i/4 & i/4 & 1/4 \end{array}\right).$$

The state vector and the density matrix of the state, where the first spin has the projection equal to +1/2 and the second spin has the projection equal to −1/2 on the *y* axis, have the form

(A8) $$|y↑,y↓\rangle =\left(\begin{array}{c} 1/2\\ -i/2\\ i/2\\ 1/2 \end{array}\right),\;\rho_{y↑,y↓}=\left(\begin{array}{cccc} 1/4 & i/4 & -i/4 & 1/4\\ -i/4 & 1/4 & -1/4 & -i/4\\ i/4 & -1/4 & 1/4 & i/4\\ 1/4 & i/4 & -i/4 & 1/4 \end{array}\right).$$

The state vector and the density matrix of the state, where the first spin has the projection equal to −1/2 and the second spin has the projection equal to +1/2 on the *y* axis, have the form

(A9) $$|y↓,y↑\rangle =\left(\begin{array}{c} 1/2\\ i/2\\ -i/2\\ 1/2 \end{array}\right),\;\rho_{y↓,y↑}=\left(\begin{array}{cccc} 1/4 & -i/4 & i/4 & 1/4\\ i/4 & 1/4 & -1/4 & i/4\\ -i/4 & -1/4 & 1/4 & -i/4\\ 1/4 & -i/4 & i/4 & 1/4 \end{array}\right).$$

The state vector and the density matrix of the state, where the first and the second spins have the projections on the *y* axis equal to −1/2, are

(A10) $$|y↓,y↓\rangle =\left(\begin{array}{c} 1/2\\ -i/2\\ -i/2\\ -1/2 \end{array}\right),\;\rho_{y↓,y↓}=\left(\begin{array}{cccc} 1/4 & i/4 & i/4 & -1/4\\ --i/4 & 1/4 & 1/4 & i/4\\ -i/4 & 1/4 & 1/4 & i/4\\ -1/4 & -i/4 & -i/4 & 1/4 \end{array}\right).$$

The state vector and the density matrix of the state, where the first spin has the projection equal to +1/2 on the *z* axis and the second spin has the projection equal to +1/2 on the *x* axis, have the form

(A11) $$|z↑,x↑\rangle =\left(\begin{array}{c} 1\\ 0 \end{array}\right)⊗\left(\begin{array}{c} 1\sqrt{2}\\ 1\sqrt{2} \end{array}\right)=\left(\begin{array}{c} 1/\sqrt{2}\\ 1/\sqrt{2}\\ 0\\ 0 \end{array}\right),\;\rho_{z↑,x↑}=\left(\begin{array}{cccc} 1/2 & 1/2 & 0 & 0\\ 1/2 & 1/2 & 0 & 0\\ 0 & 0 & 0 & 0\\ 0 & 0 & 0 & 0 \end{array}\right).$$

The state vector and the density matrix of the state, where the first spin has the projection equal to +1/2 on the *x* axis and the second spin has the projection equal to +1/2 on the *z* axis, have the form

(A12) $$|x↑,z↑\rangle =\left(\begin{array}{c} 1/\sqrt{2}\\ 1/\sqrt{2} \end{array}\right)⊗\left(\begin{array}{c} 1\\ 0 \end{array}\right)=\left(\begin{array}{c} 1/\sqrt{2}\\ 0\\ 1/\sqrt{2}\\ 0 \end{array}\right),\;\rho_{x↑,z↑}=\left(\begin{array}{cccc} 1/2 & 0 & 1/2 & 0\\ 0 & 0 & 0 & 0\\ 1/2 & 0 & 1/2 & 0\\ 0 & 0 & 0 & 0 \end{array}\right).$$

The state vector and the density matrix of the state, where the first spin has the projection equal to −1/2 on the *x* axis and the second spin has the projection equal to −1/2 on the *z* axis, have the form

(A13) $$|x↓,z↓\rangle =\left(\begin{array}{c} 1/\sqrt{2}\\ -1/\sqrt{2} \end{array}\right)⊗\left(\begin{array}{c} 0\\ 1 \end{array}\right)=\left(\begin{array}{c} 0\\ 1/\sqrt{2}\\ 0\\ -1/\sqrt{2} \end{array}\right),\;\rho_{x↓,z↓}=\left(\begin{array}{cccc} 0 & 0 & 0 & 0\\ 0 & 1/2 & 0 & -1/2\\ 0 & 0 & 0 & 0\\ 0 & -1/2 & 0 & 1/2 \end{array}\right).$$

The state vector and the density matrix of the state, where the first spin has the projection equal to −1/2 on the *z* axis and the second spin has the projection equal to −1/2 on the *x* axis, have the form

(A14) $$|z↓,x↓\rangle =\left(\begin{array}{c} 0\\ 1 \end{array}\right)⊗\left(\begin{array}{c} 1/\sqrt{2}\\ -1/\sqrt{2} \end{array}\right)=\left(\begin{array}{c} 0\\ 0\\ 1/\sqrt{2}\\ -1/\sqrt{2} \end{array}\right),\;\rho_{z↓,x↓}=\left(\begin{array}{cccc} 0 & 0 & 0 & 0\\ 0 & 0 & 0 & 0\\ 0 & 0 & 1/2 & -1/2\\ 0 & 0 & -1/2 & 1/2 \end{array}\right).$$

The state vector and the density matrix of the state, where the first spin has the projection equal to +1/2 on the *z* axis and the second spin has the projection equal to +1/2 on the *y* axis, have the form

(A15) $$|z↑,y↑\rangle =\left(\begin{array}{c} 1\\ 0 \end{array}\right)⊗\left(\begin{array}{c} 1\sqrt{2}\\ i\sqrt{2} \end{array}\right)=\left(\begin{array}{c} 1/\sqrt{2}\\ i/\sqrt{2}\\ 0\\ 0 \end{array}\right),\;\rho_{z↑,y↑}=\left(\begin{array}{cccc} 1/2 & -i/2 & 0 & 0\\ i/2 & 1/2 & 0 & 0\\ 0 & 0 & 0 & 0\\ 0 & 0 & 0 & 0 \end{array}\right).$$

The state vector and the density matrix of the state, where the first spin has the projection equal to +1/2 on the *y* axis and the second spin has the projection equal to +1/2 on the *z* axis, have the form

(A16) $$|y↑,z↑\rangle =\left(\begin{array}{c} 1/\sqrt{2}\\ i/\sqrt{2} \end{array}\right)⊗\left(\begin{array}{c} 1\\ 0 \end{array}\right)=\left(\begin{array}{c} 1/\sqrt{2}\\ 0\\ i/\sqrt{2}\\ 0 \end{array}\right),\;\rho_{y↑,z↑}=\left(\begin{array}{cccc} 1/2 & 0 & -i/2 & 0\\ 0 & 0 & 0 & 0\\ i/2 & 0 & 1/2 & 0\\ 0 & 0 & 0 & 0 \end{array}\right).$$

The state vector and the density matrix of the state, where the first spin has the projection equal to +1/2 on the *y* axis and the second spin has the projection equal to −1/2 on the *z* axis, have the form

(A17) $$|y↑,z↓\rangle =\left(\begin{array}{c} 1/\sqrt{2}\\ i/\sqrt{2} \end{array}\right)⊗\left(\begin{array}{c} 0\\ 1 \end{array}\right)=\left(\begin{array}{c} 0\\ 1/\sqrt{2}\\ 0\\ i/\sqrt{/}2 \end{array}\right),\;\rho_{y↑,z↓}=\left(\begin{array}{cccc} 0 & 0 & 0 & 0\\ 0 & 1/2 & 0 & -i/2\\ 0 & 0 & 0 & 0\\ 0 & i/2 & 0 & 1/2 \end{array}\right).$$

The state vector and the density matrix of the state, where the first spin has the projection equal to −1/2 on the *z* axis and the second spin has the projection equal to +1/2 on the *y* axis, have the form

(A18) $$|z↓,y↑\rangle =\left(\begin{array}{c} 0\\ 1 \end{array}\right)⊗\left(\begin{array}{c} 1/\sqrt{2}\\ i/\sqrt{2} \end{array}\right)=\left(\begin{array}{c} 0\\ 0\\ 1/\sqrt{2}\\ i/\sqrt{2} \end{array}\right),\;\rho_{z↓,y↑}=\left(\begin{array}{cccc} 0 & 0 & 0 & 0\\ 0 & 0 & 0 & 0\\ 0 & 0 & 1/2 & -i/2\\ 0 & 0 & i/2 & 1/2 \end{array}\right).$$

The state vector and the density matrix of the state, where the first spin has the projection equal to +1/2 on the *y* axis and the second spin has the projection equal to −1/2 on the *x* axis, have the form

(A19) $$|y↑,x↓\rangle =\left(\begin{array}{c} 1/2\\ -1/2\\ i/2\\ -i/2 \end{array}\right),\;\rho_{y↑,x↓}=\left(\begin{array}{cccc} 1/4 & -1/4 & -i/4 & 1/4\\ -1/4 & 1/4 & 1/4 & -i/4\\ i/4 & -i/4 & 1/4 & -1/4\\ -i/4 & i/4 & -1/4 & 1/4 \end{array}\right).$$

The state vector and the density matrix of the state, where the first spin has the projection equal to −1/2 on the *x* axis and the second spin has the projection equal to +1/2 on the *y* axis, have the form

(A20) $$|x↓,y↑\rangle =\left(\begin{array}{c} 1/2\\ i/2\\ -1/2\\ -i/2 \end{array}\right),\;\rho_{x↓,y↑}=\left(\begin{array}{cccc} 1/4 & -i/4 & -1/4 & i/4\\ i/4 & 1/4 & -i/4 & -1/4\\ -1/4 & i/4 & 1/4 & -i/4\\ -i/4 & -1/4 & i/4 & 1/4 \end{array}\right).$$

The state vector and the density matrix of the state, where the first spin has the projection equal to +1/2 on the *x* axis and the second spin has the projection equal to −1/2 on the *y* axis, have the form

(A21) $$|x↑,y↓\rangle =\left(\begin{array}{c} 1/2\\ -i/2\\ 1/2\\ -i/2 \end{array}\right),\;\rho_{x↑,y↓}=\left(\begin{array}{cccc} 1/4 & i/4 & 1/4 & i/4\\ -i/4 & 1/4 & -i/4 & 1/4\\ 1/4 & i/4 & 1/4 & i/4\\ -i/4 & 1/4 & -i/4 & 1/4 \end{array}\right).$$

The state vector and the density matrix of the state, where the first spin has the projection equal to −1/2 on the *y* axis and the second spin has the projection equal to +1/2 on the *x* axis, have the form

(A22) $$|y↓,x↑\rangle =\left(\begin{array}{c} 1/2\\ 1/2\\ -i/2\\ -i/2 \end{array}\right),\;\rho_{y↓,x↑}=\left(\begin{array}{cccc} 1/4 & 1/4 & i/4 & i/4\\ 1/4 & 1/4 & i/4 & i/4\\ -i/4 & -i/4 & 1/4 & 1/4\\ -i/4 & -i/4 & 1/4 & 1/4 \end{array}\right).$$
